# A Novel Retinal Ganglion Cell Promoter for Utility in AAV Vectors

**DOI:** 10.3389/fnins.2017.00521

**Published:** 2017-09-21

**Authors:** Killian S. Hanlon, Naomi Chadderton, Arpad Palfi, Alfonso Blanco Fernandez, Peter Humphries, Paul F. Kenna, Sophia Millington-Ward, G. Jane Farrar

**Affiliations:** ^1^School of Genetics and Microbiology, Smurfit Institute of Genetics, Trinity College Dublin Dublin, Ireland; ^2^Flow Cytometry Core Facility, Conway Institute, University College Dublin Dublin, Ireland; ^3^Research Foundation, Royal Victoria Eye and Ear Hospital Dublin, Ireland

**Keywords:** retina, promoter, ganglion cell, gene therapy, AAV

## Abstract

Significant advances in gene therapy have enabled exploration of therapies for inherited retinal disorders, many of which are in preclinical development or clinical evaluation. Gene therapy for retinal conditions has led the way in this growing field. The loss of retinal ganglion cells (RGCs) is a hallmark of a number of retinal disorders. As the field matures innovations that aid in refining therapies and optimizing efficacy are in demand. Gene therapies under development for RGC-related disorders, when delivered with recombinant adeno associated vectors (AAV), have typically been expressed from ubiquitous promoter sequences. Here we describe how a novel promoter from the murine *Nefh* gene was selected to drive transgene expression in RGCs. The *Nefh* promoter, in an AAV2/2 vector, was shown to drive preferential EGFP expression in murine RGCs *in vivo* following intravitreal injection. In contrast, EGFP expression from a *CMV* promoter was observed not only in RGCs, but throughout the inner nuclear layer and in amacrine cells located within the ganglion cell layer (GCL). Of note, the *Nefh* promoter sequence is sufficiently compact to be readily accommodated in AAV vectors, where transgene size represents a significant constraint. Moreover, this promoter should in principle provide a more targeted and potentially safer alternative for RGC-directed gene therapies.

## Introduction

In recent years significant progress has been made in gene therapy with the market authorisation of therapies such as Glybera®, T-VEC (Imlygic™), and Strimvelis™. Many more potential gene therapies are currently in later phase clinical trials (clinicaltrials.gov) and it is likely that the rate of clinical development will continue to increase. Advances in our understanding of viral vectors has allowed for the generation of a range of delivery vehicles that can collectively target a wide array of cell types.

The retina in particular has been the focus of many gene therapy studies, stimulated in part by the elucidation of the genetic pathogenesis of inherited retinal degenerations (Farrar et al., 2017). The retina is a confined but readily accessible target, and retinal neurons are non-dividing in mammals—thus a gene therapy can in theory provide long-term benefit. Furthermore the retina is immune privileged and therefore, in principle, may be more tolerant of treatments. Indeed, over 30 gene therapy clinical trials have been completed or are ongoing in the retina (clinicaltrials.gov). Adeno-associated virus (AAV) has been the vehicle of choice for the majority of retinal gene therapy studies as it achieves efficient neuronal transduction, provides long-term expression in terminally differentiated cells and has demonstrated a good safety profile in humans (Hauswirth et al., 2008; MacLaren et al., 2014; Bainbridge et al., 2015; Bennett et al., 2016; Feuer et al., 2016; Ghazi et al., 2016; clinicaltrials.gov). The successful completion of a Phase III trial to treat RPE65-linked inherited retinal degenerations, such as Leber congenital amaurosis (LCA, www.sparktx.com) represents a significant milestone in the field.

Many therapeutic studies to date targeting the retina have been directed toward photoreceptor cells and the retinal pigment epithelium (RPE). Approximately 1/3000 people worldwide suffer from an inherited retinal degeneration (IRD); many of these are caused by mutations directly or indirectly affecting photoreceptors (Bessant et al., 2001). However, retinal disorders involving the ganglion cell layer (GCL) will also be readily amenable to gene therapy, given efficient cell targeting. Intravitreal injection of AAV for delivery to GCL typically involves less surgical trauma than subretinal injection. Notably, anti-VEGF treatments, such as Lucentis, are routinely administered intravitreally to age related macular degeneration (AMD) patients.

Limiting expression of a gene therapy to a target cell type is often preferable, and in principle represents a valuable safety feature. Although AAV-mediated ocular gene therapy has been shown to be well-tolerated (Hauswirth et al., 2008; MacLaren et al., 2014; Bainbridge et al., 2015; Bennett et al., 2016; Feuer et al., 2016; Ghazi et al., 2016), directing transgene expression to the target cells of interest may reduce the chance of immune response(s) or other unwanted off-target effects, thus providing a more efficacious therapy. A number of gene therapies have been directed toward retinal ganglion cells (RGCs) using AAV serotype 2, with several ongoing or completed clinical trials (Feuer et al., 2016; Yang et al., 2016; clinicaltrials.gov). Such therapies have typically utilized ubiquitous promoters such as cytomegalovirus (*CMV*) or chicken-β-actin (*CBA*; Boye et al., 2010; Koilkonda et al., 2010; Bennett et al., 2016; Feuer et al., 2016). These promoters typically offer high levels of expression, and tend to be small in size, which is valuable as the packaging capacity of AAV is limited to approximately 2–5 kb, with an optimum at 4.7 kb (Dong et al., 1996; Grieger and Samulski, 2005). However, a significant disadvantage of generic promoters is that, they may drive gene expression in cell types other than the target cells. Cell-type specific promoters such as rhodopsin (Flannery et al., 1997; Bennett et al., 1998; O'Reilly et al., 2007; Palfi et al., 2010; Wert et al., 2013), rhodopsin kinase (Khani et al., 2007; Boye et al., 2010; Sun et al., 2010; Kay et al., 2013; Molday et al., 2013), *RPE65* (Bainbridge et al., 2008, 2015) and retinaldehyde binding protein 1 (*RLBP1*; Choi et al., 2015), among others, have successfully been used in retinal gene therapy approaches. Preferential RGC expression in transgenic animal models has been achieved using the *Thy1* promoter, which confers high-level expression that is limited to RGCs. It has been shown that an enhancer element contained in the first intron of *Thy1* is necessary for both high level and specific gene expression (Spanopoulou et al., 1991; Alić et al., 2016). However, while the core promoter and enhancer element are both small (~100–200 bp each), approximately 6 kb of spacing between the two elements is necessary for specific promoter function, making the *Thy1* promoter unsuitable for use in AAV vectors. A 0.48 kb promoter derived from the human synapsin-1 gene (hSYN) can provide pan-neuronal expression in rodent and primate brains when utilized in adenoviral or AAV vectors (Kügler et al., 2003a,b; Diester et al., 2011; Lopez et al., 2016). In the rodent retina, intravitreal injection of an AAV gene construct driven by hSYN resulted in expression in the GCL (Gaub et al., 2014). However, in the context of the primate retina, hSYN promoter-mediated expression only appears to occur in damaged retinas or vitreolysed eyes (Yin et al., 2011). The therapeutic relevance of the hSYN promoter therefore remains to be fully established. Hence, the characterization of a promoter that exhibits preferential RGC expression and is appropriately sized for AAV would represent a significant refinement for RGC gene therapies.

Increasingly, RGC subtypes are being defined by differential gene expression, rather than morphological differences. Several groups have worked on establishing the transcriptional differences between different classes of RGCs using immunological, transcriptomic and transgenic methods (Sanes and Masland, 2014; Sun et al., 2015; Rousso et al., 2016; Sweeney et al., 2017). There are approximately 1.5 million RGCs in the human retina, comprising approximately 1% of retinal neurons, (Callaway, 2005) and composed of in the region of 30 different classes of cells (Baden et al., 2016). However, knowledge regarding the different types of RGCs populating the GCL is still emerging. Transcriptomics offers a powerful means to analyse gene expression in different cell types. In order to identify potential RGC promoters, GCL-specific microarray expression data from post-mortem human retinas was used (Kim et al., 2006). Kim et al. isolated GCL populations consisting of 1,000 RGCs using laser-capture microdissection (LCM) and cell populations consisting of 1,000 cells from the remainder of the retina (termed outer retina, OR) and compared gene expression in the two populations. Using these data, we assessed promoter conservation between mammalian species for genes that were highly expressed and enriched in RGCs, using data drawn from the UCSC database (mm10; Kent et al., 2002). Conservation of non-coding DNA sequence across species was used as an indicator of potential function, and a number of highly conserved promoter upstream sequences were identified from genes shown to be both highly expressed and enriched in RGCs (Kim et al., 2006; Choudhury et al., 2016; Struebing et al., 2016). The lead candidate promoter, upstream of the *Nefh* gene, was evaluated and compared to *CMV*-driven gene expression in RGCs *in vivo*. The *Nefh* promoter directed expression preferentially to RGCs when administered intravitreally to adult wild type mice using AAV2, in contrast to the broad expression pattern observed with the *CMV* promoter. The study identifies a functional RGC promoter, which is suitable for AAV-mediated ocular gene delivery, while also describing an approach to identify putative promoters.

## Materials and methods

### *In silico* RGC promoter analyses

**Figure 1:**
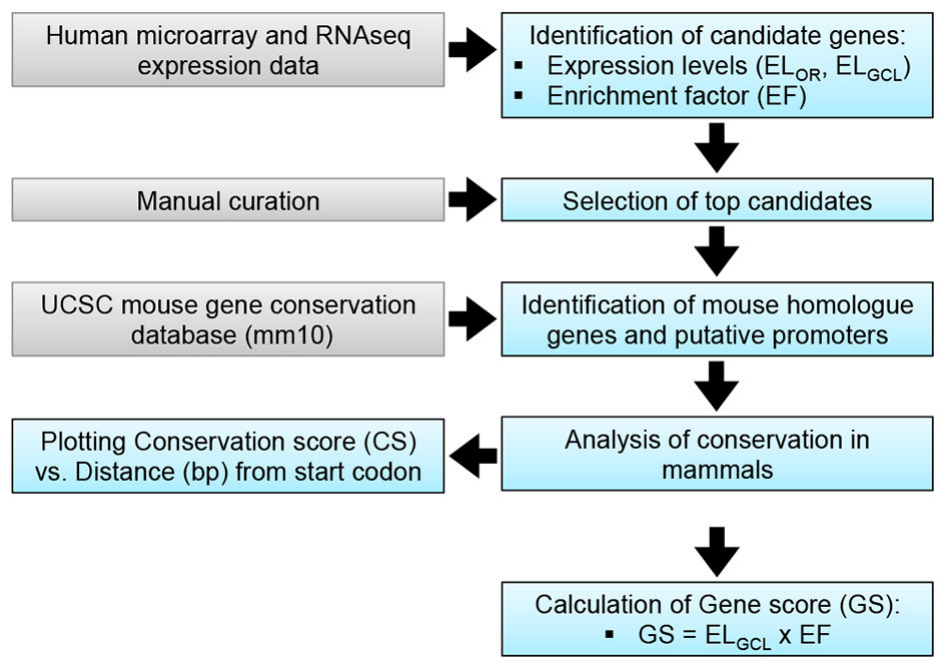
RGC promoter analysis. A: Putative promoter identification methodology. Transcriptomic data (Kim et al., 2006) was used to identify candidate genes, based on expression levels in the retina (EL_OR_) and the GCL (EL_GCL_). Enrichment factor (EF) for the GCL was calculated as EF = EL_GCL_/EL_OR_; top candidates were identified based on EF. A gene score (GS) was calculated as a means of discerning between candidates.

**Figure 2:**
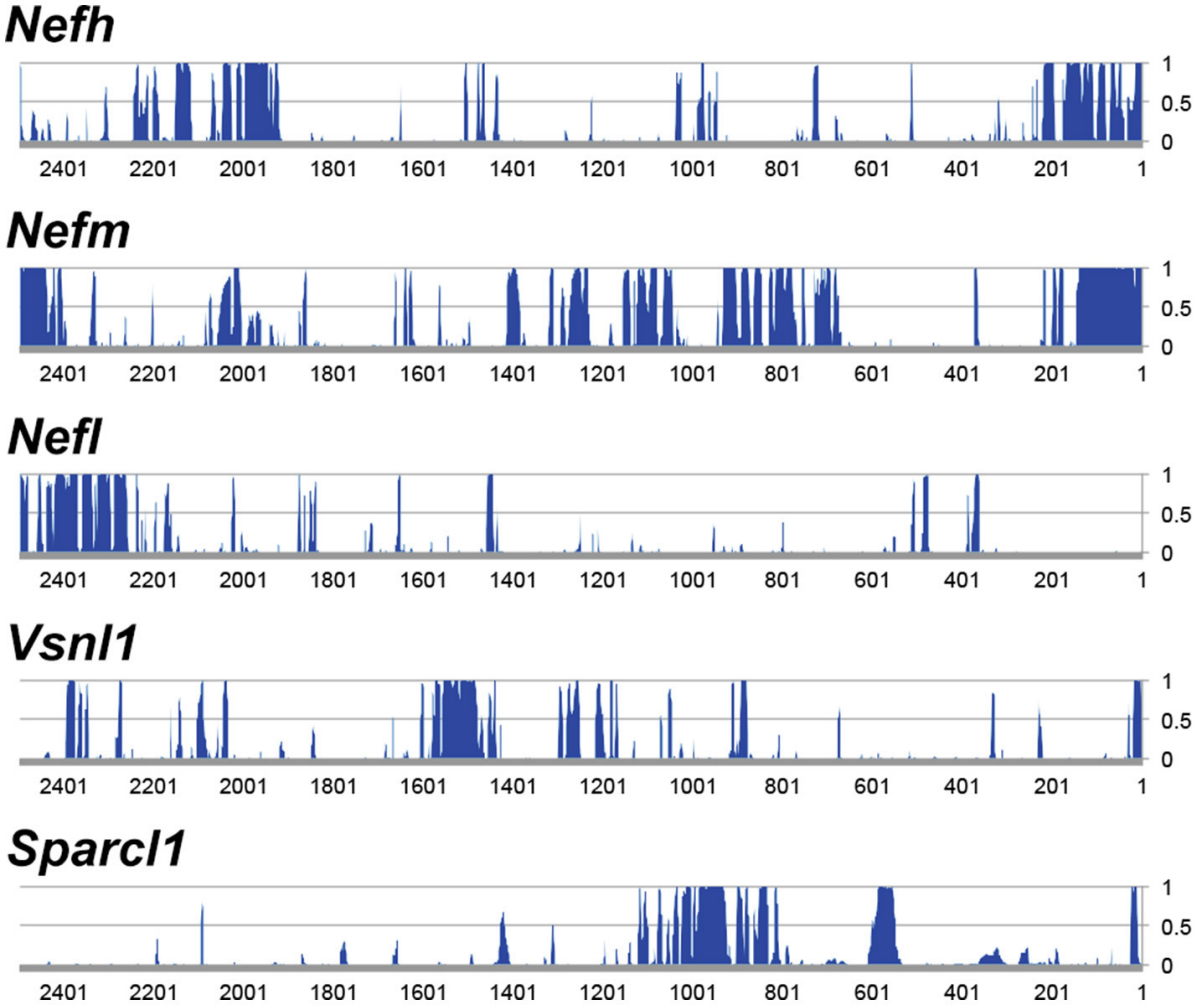
Analysis of 5′ upstream sequence of candidate promoter sequences. Regions ~2.5 kb upstream of the transcriptional start were analyzed. The genes displayed represent the most highly expressed genes of the genes that are enriched >10-fold in RGCs. The y-axis represents conservation across mammals (CS), where 0 equals no significant conservation and 1 equals full conservation across mammalian species in the UCSC genome database.

Human genes whose relative expression was enriched in the RGC layer by over 10-fold compared to relative expression in OR were selected (Kim et al., 2006). Genes were assessed based on GCL expression level (EL_GCL_) compared to the OR expression (EL_OR_), termed the enrichment factor (EF = EL_GCL_/ EL_OR_; Kim et al., 2006) and the genes with the highest EFs were selected for further investigation. A gene score (GS = EL_GCL_ × EF) was used to rank genes for suitability as potential promoters. Further analysis was performed on mouse genomic data, as a mouse promoter was the desired output. Data from the UCSC genome browser (mm10 mouse mammalian conservation track; UCSC; Kent et al., 2002) were used to establish conservation upstream of the transcriptional start site of candidate genes; results from analysis of 2.5 kb upstream of the start site are presented (Figures 1, 2). An *in silico* pipeline (Python) was developed to isolate basewise conservation data from UCSC (conservation data ranged from 0 to 1 for a given base, where 0 represents no significant conservation between mammals and 1 indicates complete conservation). The forty mammalian species and their sequence assembly names that make up this conservation data are listed in Table S1. This was plotted in a graph in order to visualize conserved regions. *NEFH* was chosen as having the highest GS of the genes analyzed. Using the parameters defined above, the mouse *Nefh* upstream region was selected for evaluation *in vivo*, given the expression profile of the gene and conservation of its 5′ upstream sequence.

### Cloning and AAV production

pAAV.*CMV*-EGFP was cloned as described (Palfi et al., 2010). To generate pAAV.*Nefh*-EGFP, a 2,251 bp fragment of mouse *Nefh* upstream sequence (NM_010904.3) was amplified from genomic DNA and were substituted for the *CMV* promoter in pAAV.*CMV*-EGFP. Recombinant AAV2/2 viruses AAV.*Nefh*-EGFP and AAV.*CMV*-EGFP were generated, and genomic titres determined as described (O'Reilly et al., 2007).

### Animals and intravitreal injections

Wild type 129 S2/SvHsd mice (Harlan UK Ltd, Oxfordshire, UK) were maintained in a specific pathogen free (SPF) facility. Intravitreal injections were undertaken in strict compliance with the European Communities Regulations 2002 and 2005 (Cruelty to Animals Act) and the Association for Research in Vision and Ophthalmology (ARVO) statement for the use of animals. Adult mice were anesthetized and pupils dilated as described (O'Reilly et al., 2007). Using topical anesthesia (Amethocaine), a small puncture was made in the sclera. A 34-gauge blunt-ended microneedle attached to a 10 μl Hamilton syringe was inserted through the puncture, and 3 μl AAV2/2 was slowly, over a 2-min period, administered into the vitreous. Following intravitreal injection, an anesthetic reversing agent (100 mg/10 g body weight; Atipamezole Hydrochloride) was delivered by intraperitoneal injection. Body temperature was maintained using a homeothermic heating device. Animals were sacrificed by CO_2_ asphyxiation.

### Histology

Histology was performed as described (Chadderton et al., 2013) with some modifications. Briefly, transduced eyes (*n* = 6) were fixed in 4% paraformaldehyde and cryosectioned (12 μm). Sections were co-labeled for EGFP (chicken anti-GFP; Abcam, ab13970, 1/2000 dilution; Palfi et al., 2012) and either Brn3a (Nadal-Nicolás et al., 2009; Trost et al., 2015; Santa Cruz Biotechnology, sc-31984, 1/200 dilution; goat anti-Brn3a), ChAT (goat anti-choline acetyltransferase; Millipore, AB144P, 1/500 dilution; Zhu et al., 2014) or GABA (rabbit anti-GABA; Sigma, A2052, 1/2000 dilution; Zhu et al., 2014) using immunohistochemistry. EGFP was labeled with FITC-conjugated secondary antibody (1/400 dilution, Jackson ImmunoResearch Laboratories) while Brn3a, ChAT and GABA were labeled with Cy3-conjugated secondary antibody (1/400 dilution, Jackson ImmunoResearch Laboratories). Cell nuclei were counterstained with 4,6-diamidino-2-phenylindole (DAPI). Background labeling was determined using parallel processed sections where the primary antibodies were omitted. Corresponding microscope images were taken using a Zeiss Axiophot fluorescent microscope (Carl Zeiss Ltd., Welwyn Garden City, UK). Immunohistochemical signals obtained with different filters were overlaid using Photoshop v.13 (Adobe Systems Europe, Glasgow, UK). For analysis, levels for each channel were set to predetermined values to help discrimination between signal and background; signal levels above threshold were taken as positive. Additionally, cellular colocalisation of the positive immunohistochemical signals with the nuclear label was a criterion for identification of positive cells. However, it is possible that at the low spectrum identification of either positive or negative cells failed. This would have implicated a small percentage of cells and affected all groups similarly, therefore should not have any significant effects on the results. Labeled and co-labeled cells were counted manually using the count tool in Photoshop. Two transduced sections (approximately 300 μm apart) from the central part of the retina (~1,500 μm span in total) were analyzed for each marker (*n* = 4–5). Statistical analysis (one way ANOVA, Tukey's multiple comparison *post-hoc* test) was performed using Prism 5 (GraphPad); *p* < 0.05 was considered statistically significant.

### Flow cytometry cell sorting

Retinas were harvested 3 weeks post-injection and trypsin-dissociated, as previously described (Palfi et al., 2012). To isolate RGCs, cells were labeled with anti-Thyl-PE-Cy5, (CD90.2, Rat Thy1.2, 53-2.1 1:100; eBioscience Inc., San Diego, CA). DRAQ5^TM^ (BioStatus, Leicestershire, UK) was used to sort nucleus-positive cells after which cell populations were sorted on the basis of forward and sideways scatter, and subsequently two stages of singlet selection. From these, retinal cells expressing both EGFP and Thy1 were identified (BD FACSAria IIIu high speed cell sorter, BD Bioscience, San Jose, CA). EGFP was excited by a 488 nm laser and the emission was collected using a 530/30 band pass filter. Thy-1 PECy5 had been measured exciting the probe with a 561 nm laser and collecting the signal with a 690/40 nm band pass. QC of the cell sorter had been done with BD CS&T beads and the drop delay had been adjusted using the BD Accudrop beads (RUO), following manufacture specifications. EGFP-positive cells expressing Thy1 were represented as a percentage of the total EGFP positive cells. Data has been reanalyzed with the FCSExpress 6 Flow software (DeNovo Software). Statistical analysis (Student's *t*-test) was performed using Microsoft Excel and *p* < 0.05 was considered statistically significant.

### RT-qPCR

Thy1-positive cells collected from *n* = 12 retinas and non-labeled retinal cells with a similar forward and side scatter from *n* = 9 retinas were collected by flow cytometry and total RNA was extracted as described (Millington-Ward et al., 2011). *Thy1* mRNA was amplified in triplicate from pooled sorted populations using the QuantiTect SYBR green RT-PCR kit (Qiagen, Hilden, Germany) using the manufacturer's protocol and the following primers:

F 5′ TGAACCAAAACCTTCGCCTG 3′
R 5′ AGCTCACAAAAGTAGTCGCC 3′

Resulting CT values were standardized to cell number, as standardly used housekeeping genes could be expressed at different levels in different cell populations, making them unreliable for this analysis.

## Results

The objective of the current study was the characterization and *in vivo* evaluation of an RGC promoter for future use in AAV-mediated gene therapies. A comparative evaluation of genes with highly enriched RGC expression was undertaken *in silico* and the lead candidate was investigated *in vivo* (Figure 1). Whilst gene expression profiles of RGCs are available, the promoters that drive this expression are ill defined. We chose several key criteria to identify candidate promoters using microarray data for RGCs (Kim et al., 2006; Choudhury et al., 2016; Struebing et al., 2016). Conservation data of regions upstream of the most enriched RGC candidate genes were obtained from the UCSC genome browser database (UCSC, mm10). In the study conservation of sequence across mammals (using the mouse genome as a base) was used as a proxy for presumed function *in vivo* to identify putative promoters. To ensure that any promoter chosen would be suitable for future use in AAV vectors, conservation analysis was limited to the immediate 2.5 kb upstream sequence of genes. Based on the expression level of a gene in the GCL (EL_*GCL*_) and the enrichment factor of that gene (EF), a gene score was generated to rank genes as candidates (GS = EL_*GCL*_ × EF; Table 1). The basewise species conservation in the selected upstream sequences was plotted (conservation numbered between 0 and 1) and the five genes with the highest GS are presented (Figure 2).

**Table 1:** List of putative ganglion cell promoters.

**Rank**	**Gene name**	**EL_GCL_**	**EL_OR_**	**EF**	**GS**
1	*NEFH*	21,899.1	89.4	245	5.37 × 10^6^
2	*NEFM*	6,984.1	31.7	220.6	1.54 × 10^6^
3	*NEFL*	7,841.1	50.5	155.3	1.22 × 10^6^
4	*VSNL1*	4,659.33	67.35	69.18	3.22 × 10^5^
5	*SPARCL1*	5077	149.75	33.9	1.72 × 10^5^
6	*SLC17A6*	1,302.9	10.3	126.8	1.65 × 10^5^
7	*TMSB10*	7,124.3	324.6	21.9	1.56 × 10^5^
8	*ANXA2*	2,221.4	37.5	59.3	1.32 × 10^5^
9	*STMN2*	4,139.9	147.9	28	1.16 × 10^5^
10	*PRPH1*	1,238.5	18.4	67.5	8.36 × 10^4^
11	*CRTAC1*	4,478.6	347	12.9	5.78 × 10^4^
12	*RBPMS*	832.5	12.6	66	5.49 × 10^4^
13	*RAB13*	1,802.7	59.7	30.2	5.44 × 10^4^
14	*ATP1B1*	3,803.3	299.2	12.7	4.83 × 10^4^
15	*FABP3*	1,054.6	24.9	42.4	4.47 × 10^4^

*Human transcriptomic data of 1,000 cell populations from RGCs versus OR (Kim et al., 2006) was used to determine relative expression levels in the outer retina (EL_OR_) and the GCL (EL_GCL_). Enrichment factor (EF) for the GCL was calculated as EF = EL_GCL_/EL_OR_. A gene score (GS) was calculated as GS = (EL_GCL_ x EF) to provide an overall score. Genes are listed in order of GS*.

Following analysis, *Nefh* was deemed to be the most highly enriched gene in RGCs with an enrichment factor (EF) of 245-fold, as well as demonstrating an extremely high EL_GCL_ (21,899.1; Table 1). Some of the mouse genes analyzed showed greater average conservation in their 2.5 kb upstream regions than *Nefh* (*Nefm* 0.289, *Stmn2* 0.292, *Crtac1* 0.349 vs. *Nefh* 0.185). However, due to their lower EF and EL_*GCL*_ scores, *Nefh* was deemed likely to drive higher levels of RGC-specific expression and hence to be a better candidate promoter (GS: *Nefh* 5.37 × 10^6^ vs. *Nefm* 1.54 × 10^6^ *Stmn2* 1.16 × 0^6^, *Crtac1* 5.78 × 10^4^). *Tmsb10, Nefl*, and *Sparcl1* had lower scores than *Nefh* in all categories. *Brn3a*, a commonly used marker for RGCs (Kim et al., 2006; Nadal-Nicolás et al., 2014), was found to have an extremely high conservation within a 2.5 kb upstream region, and a high EF (0.576, 79.1 respectively). However, its EL_GCL_ was found to be approximately 39 times lower than that of *Nefh* (719.7), and so was not included as a candidate gene. The *hSYN* gene showed no significant GCL enrichment or expression in the Kim et al. (2006) study.

To explore the strength and specificity of the putative *Nefh* promoter, 2,251 bp of upstream sequence from the mouse homolog was used to drive expression of an *EGFP* reporter gene in an AAV2/2 vector (AAV-*Nefh*-EGFP) and expression compared to that mediated by the *CMV* promoter (AAV-*CMV*-EGFP; Chadderton et al., 2013; Palfi et al., 2015). The mouse gene was chosen to ensure that function or non-function was not due to species incompatibility. The *CMV* promoter incorporated into AAV vectors has previously been shown to drive high levels of transgene expression in a wide variety of retinal cell types (Lebherz et al., 2008; Li et al., 2008; Mueller and Flotte, 2008), including RGCs (Chadderton et al., 2013; Tshilenge et al., 2016) and was used as a control vector for transgene expression.

**Figure 3:**
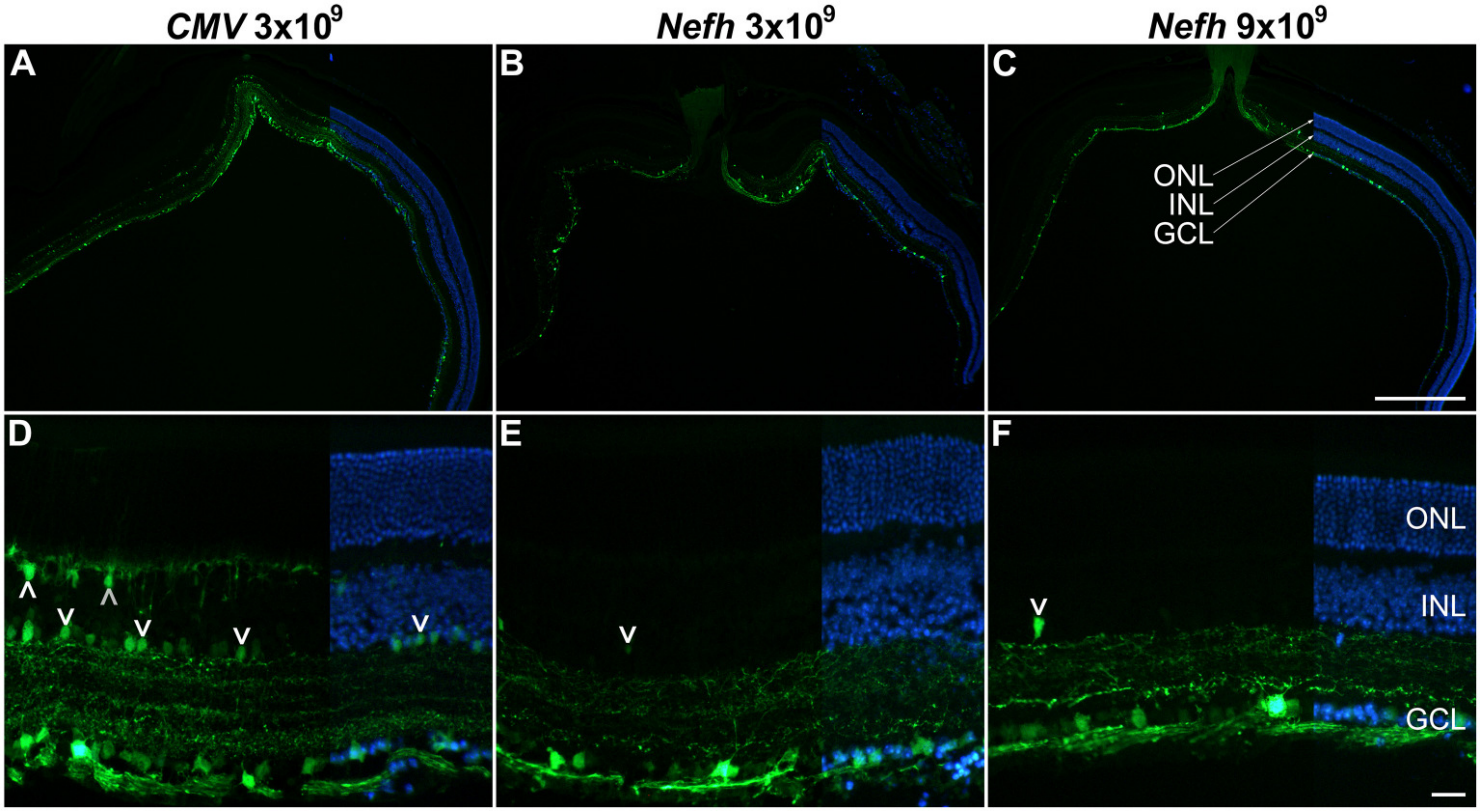
Comparison of *CMV* and *Nefh* mediated EGFP expression *in vivo*. Retinas were injected intravitreally with AAV.*CMV*-EGFP (3 × 10^9^ vg; **A,D**) or two different doses of AAV.*Nefh*-EGFP (3 × 10^9^ vg; **B,E**; and 9 × 10^9^ vg; **C,F**). Transduced eyes (*n* = 4–5) were fixed and cryosectioned 12 weeks post-delivery. FITC-labeled immunocytochemistry was performed for EGFP. DAPI was used for nuclear counterstaining; DAPI signals are overlaid on the right side of the images. ONL, outer nuclear layer; INL, inner nuclear layer, GCL, ganglion cell layer. Arrowheads: transduced cells in the INL. Scale bars: 500 μm **(C)** and 25 μm **(F)**.

Adult mice were injected intravitreally with 3 × 10^9^ viral genomes (vg)/eye AAV.*CMV*-EGFP or with either 3 × 10^9^ vg/eye or 9 × 10^9^ vg/eye AAV.*Nefh*-EGFP. Histological analysis 12 weeks post-injection revealed widespread EGFP expression in the retina (Figure 3). Individual cells exhibited a broad range of EGFP expression levels from low to very high, possibly due to varying viral transduction. However, cellular EGFP labeling (colocalised to DAPI stained nuclei), even for cells expressing low levels of EGFP, was easily distinguishable from uniform background levels. EGFP expression from both promoters was observed in a significant number of cells in the GCL (50.2 ± 14.1% AAV.*CMV*-EGFP, Figures 3A,D; 42 ± 11.2% AAV.*Nefh*-EGFP, Figures 3B,E; and 37 ± 11.1% high dose AAV.*Nefh*-EGFP, Figures 3C,F, 5A). However, while the *Nefh* promoter mediated EGFP expression was predominantly confined to the GCL (Figures 3B,C,E,F), *CMV* promoter driven expression extended into the INL (Figures 3A,D); 84.5 ± 34.2% AAV.*CMV*-EGFP, 3.6 ± 2.9% AAV.*Nefh*-EGFP and 5.6 ± 3.8% high dose AAV.*Nefh*-EGFP (Figure 5A; EGFP-positive cells in the INL expressed as a percentage of all cells in the GCL). Notably, the increased dose of AAV.*Nefh*-EGFP did not increase the transduction rate in the INL (Figures 3, 5A). AAV.*CMV*-EGFP demonstrated significantly greater INL expression compared to AAV.*Nefh*-EGFP, *p* < 0.001 (Figures 3, 5A).

**Figure 4:**
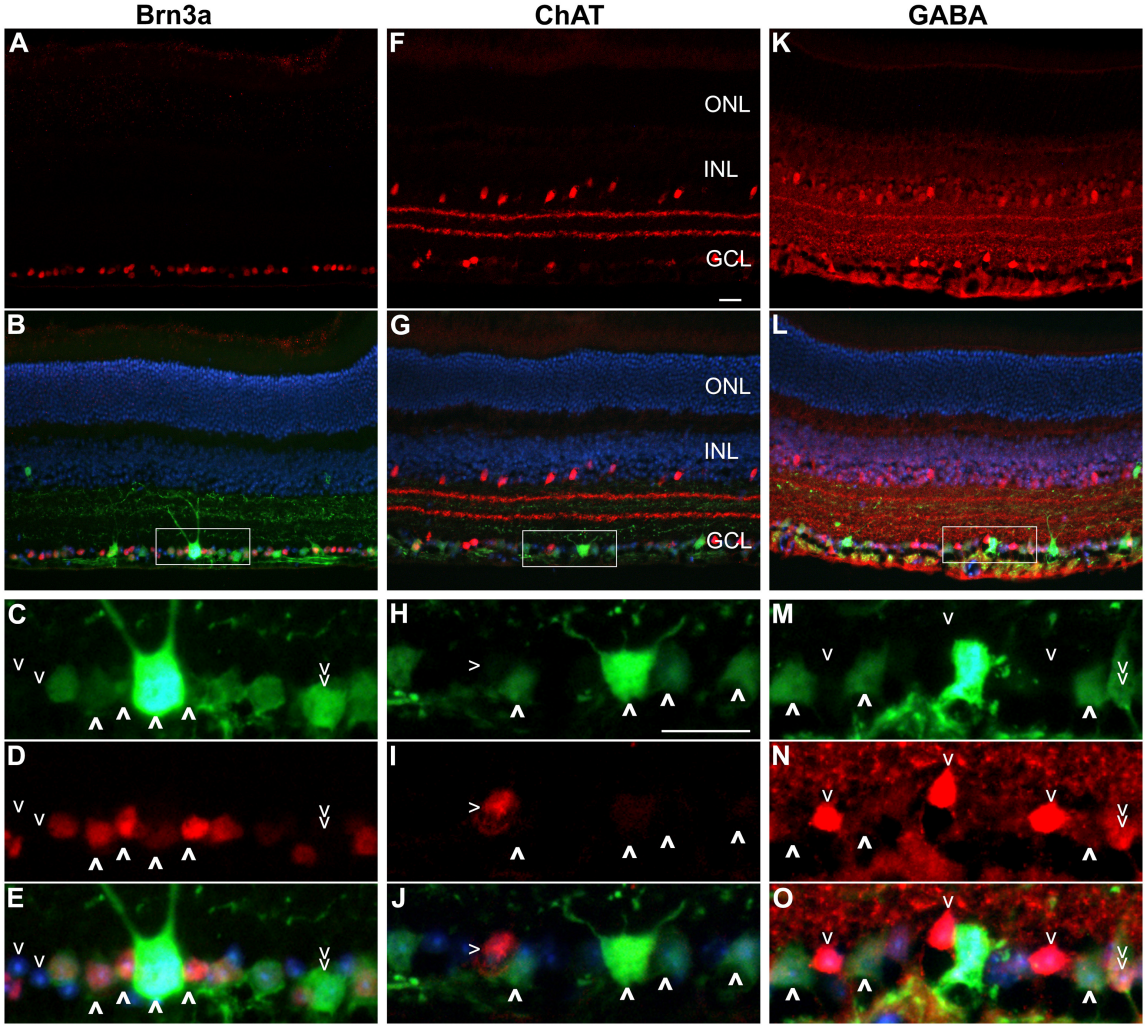
Immunocytochemistry analysis of AAV.*Nefh*-EGFP transduced retinas. Eyes were injected intravitreally with AAV.*Nefh*-EGFP (3 × 10^9^ vg). Transduced eyes (*n* = 5) were fixed and cryosectioned 12 weeks post-delivery. Immunocytochemistry was performed for Brn3a (Cy3; **A–E**), ChAT (Cy3; **F–J**) and GABA (Cy3; **K–O**) in combination with EGFP labeling (FITC). DAPI was used for nuclear counterstaining. Rectangles (in **B,G,L**) indicate positions of the enlarged areas. **A,F,K**: Cy3 label; **B,G,L**: Cy3, FITC and DAPI overlaid. **C,H,M**: FITC label; **D,I,N**: Cy3 label; **E,J,O**: Cy3, FITC and DAPI labels overlaid. (**C–E**) Bold arrowheads: transduced Brn3a-positive cells. Regular arrowheads: un-transduced Brn3a-negative cells. Double arrowhead: a transduced Brn3a-negative cell. **(H–J)** Bold arrowheads: transduced ChAT-negative cells. Regular arrowhead: a transduced ChAT-positive cell. **(M–O)** Bold arrowheads: transduced GABA-negative cells. Regular arrowheads: un-transduced GABA-positive cells. Double arrowhead: an un-transduced GABA-positive cell. ONL, outer nuclear layer; INL, inner nuclear layer, GCL, ganglion cell layer. Scale bars: 25 μm **(F,H)**.

**Figure 5:**
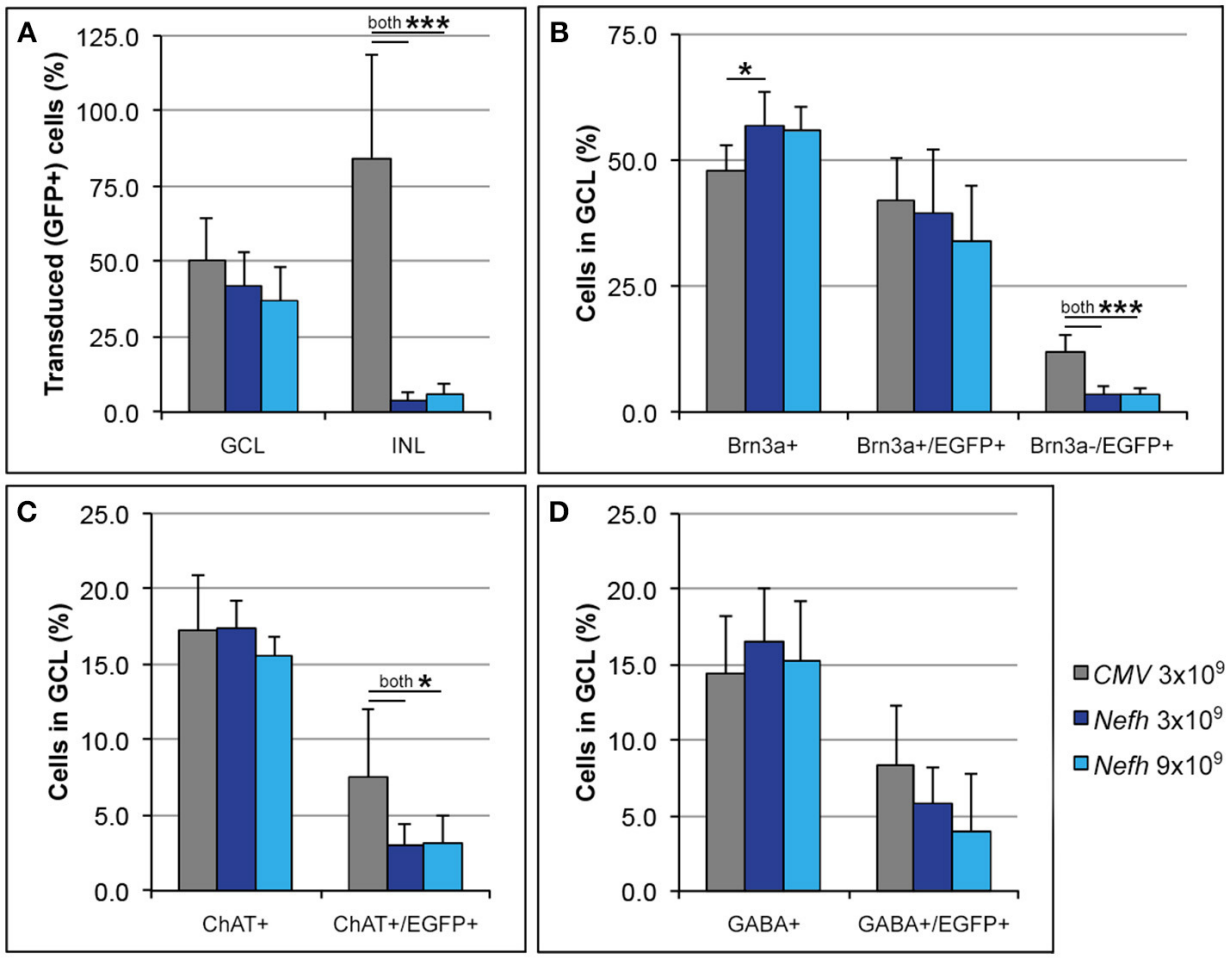
Quantification of *CMV* and *Nefh* mediated EGFP expression *in vivo*. Retinas were injected intravitreally with AAV.*CMV*-EGFP (3 × 10^9^ vg; **A,D**) or two different doses of AAV.*Nefh*-EGFP (3 × 10^9^ vg; **B**; and 9 × 10^9^ vg; **C**). Transduced eyes (*n* = 4–5) were fixed and cryosectioned 12 weeks post-delivery. Immunocytochemistry was performed for Brn3a, ChAT, GABA, and EGFP; DAPI was used for nuclear counterstaining. Manual quantification of labeled and co-labeled cells was performed in the immunolabelled retinal sections. **(A)** Distribution of EGFP positive cells was determined in the ganglion cell (GCL) and the inner nuclear layers (INL). Additionally, co-localization of EGFP with Brna3a **(B)**, ChAT **(C)**, and GABA **(D)** was determined in the GCL. ^***^p < 0.001; ^*^p < 0.05 (ANOVA).

Approximately fifty percent of cells in the GCL are RGCs with the other 50% being displaced amacrine cells (Jeon et al., 1998; Akopian et al., 2016; webvision.med.utah.edu). To delineate further the expression profile of the *Nefh* promoter, EGFP transgene expression was analyzed in the GCL using antibodies targeting Brn3a, an RGC marker (Schlamp et al., 2013) and two amacrine cell markers, ChAT and GABA (Figure 4) (Wässle et al., 1987; Jeon et al., 1998; webvision.med.utah.edu). Brn3a label was confined to the GCL in the retina and was used to explore the specificity of the putative *Nefh* promoter for RGCs (Figures 4A–E). In line with previously published data, 50–55% of all cells in the GCL were Brn3a positive (Figures 4, 5B; Jeon et al., 1998; Schlamp et al., 2013). Figure 4 displays representative images from eyes injected with the 3 × 10^9^ vg/eye dose of AAV.*Nefh-EGFP*. While AAV.*CMV*-EGFP and AAV.*Nefh*-EGFP expressed in comparable numbers of Brn3a positive cells (41.9 ± 8.5% AAV.*CMV*-EGFP, 39.5 ± 12.7% AAV.*Nefh*-EGFP and 33.9 ± 11.2% high dose AAV.*Nefh*-EGFP; Figures 4, 5B), *Nefh* promoter-mediated EGFP expression in the GCL was observed in significantly fewer Brn3a negative cells (*p* < 0.001, 12.1 ± 3.3 AAV.*CMV*-EGFP, 3.5 ± 1.7 AAV.*Nefh*-EGFP and 3.4 ± 1.2 high dose AAV.*Nefh*-EGFP; Figures 4, 5B). ChAT (Figures 4F–J) and GABA (Figures 4K–O) markers were used to analyse subpopulations of amacrine cells. ChAT labeled cells in both the INL and GCL, resulting in prominent “double-layered” staining within the IPL (Figures 4F–J); ChAT labeling identified approximately 17% cells in the mouse GCL (Figure 4, 5C), in line with previous data (Crooks and Kolb, 1992). GABA immunostaining resulted in widespread labeling in the mouse retina (Figures 4K–O); GABA labeling identified approximately 15% of cells in the GCL in our study (Figure 4, 5D). EGFP expressing cells were significantly more likely to be ChAT positive amacrine cells (Figures 4F–J) when EGFP expression was driven by the *CMV* promoter, compared to the *Nefh* promoter (*p* < 0.05, 7.5 ± 4.6% AAV.*CMV*-EGFP, 3.1 ± 1.4% AAV.*Nefh*-EGFP and 3.2 ± 1.9% high dose AAV.*Nefh*-EGFP; Figure 5C). Additionally a greater number of *CMV* promoter driven EGFP positive cells were also co-labeled with GABA, however this represented a trend rather than reaching significance (8.3 ± 4.0% AAV.*CMV*-EGFP, 5.8 ± 2.4% AAV.*Nefh*-EGFP and 4.0 ± 3.7% high dose AAV.*Nefh*-EGFP; Figure 5D).

As a second method of assessing preferential gene expression in RGCs from the *Nefh* promoter, adult wildtype mice were intravitreally injected with 9 × 10^9^vg/eye AAV.*Nefh*-EGFP or 3 × 10^9^vg/eye AAV.*CMV*-EGFP. Three weeks post injection, retinas were taken, cells dissociated and analyzed by cell sorting and EGFP-positive cells assessed for Thy1 expression. Interestingly levels of Thy1 enrichment in these populations were significantly higher in AAV.*Nefh*-EGFP versus AAV.*CMV*-EGFP transduced retinal samples (5.4-fold, *n* = 12 vs. only 1.6-fold, *n* = 9 respectively; *p* < 0.005). These data support the immunohistochemical observations above. Notably, *Thy1* mRNA levels were found to be 3.23-fold higher in Thy1-positive cells than in non-antibody labeled retinal cells with a similar forward and sideways scatter (CT values of 32.618 and 33.477 respectively), indicating that the Thy1 antibody enriches for RGCs (Figure S1).

## Discussion

AAV has become one of the most commonly used vectors for gene therapy, with many clinical trials ongoing or completed and a number of gene therapies approved or seeking approval (clinicaltrials.gov). AAV is also the predominantly used vector in ocular gene therapies, with AAV2 currently the serotype of choice for RGC directed approaches (Hauswirth et al., 2008; MacLaren et al., 2014; Bainbridge et al., 2015; Bennett et al., 2016; Feuer et al., 2016; Ghazi et al., 2016; Yang et al., 2016; clinicaltrials.gov). Research in recent years has focused on improving the efficiency of AAV transduction and expression in the retina. The development of AAV vectors such as AAV7m8 and AAV8BP2 has improved levels of transduction in a wide variety of retinal cell types, and enabled consideration of intravitreal administration as a potential route of access to many retinal cells including photoreceptors (Dalkara et al., 2013; Cronin et al., 2014; Ramachandran et al., 2016). Various tyrosine capsid mutations in AAV have the potential to increase transgene expression levels by modulating capsid phosphorylation and ubiquitin proteasome-based degradation of viral particles during intracellular trafficking (Petrs-Silva et al., 2009; Mowat et al., 2014; Mao et al., 2016). Recent approaches to intravitreal delivery, including vitrectomy and sub-inner limiting membrane (sub-ILM) blebbing, have the potential to improve expression levels further (Boye et al., 2010; Tshilenge et al., 2016). However, a consequence of more efficient and broad transduction profiles may be greater potential for off-target effects. Confining expression of a gene therapy to only those cells affected by a disease represents a rational strategy; the potential reduction in immune responses may be an advantageous safety feature, as well as a means of aiding long-term expression.

In the current study, we have developed an approach to identify putative RGC promoters by analyzing retinal transcriptomic data and referencing it against mammalian sequence conservation datasets to infer potential function. The expression levels of retinal genes were analyzed, with high GCL enrichment and high absolute expression levels prioritized. Gene expression data in RGCs from the gene expression omnibus (GEO; ncbi.nlm.nih.gov/geo) was analyzed in detail. Studies on expression from pre-natal or immature retina were omitted. In addition, samples where photoreceptor cell-specific gene expression was found to be high in RGCs were excluded as this indicated sample impurity. In contrast to the data from Kim et al. (2006), and taking the above into account, no other studies in the database suitably provided data on RGC gene expression enrichment in adult retina.

Conservation of the upstream sequence of these genes was evaluated in this context in order to establish lead candidate promoter sequences. Using this approach, we identified a number of potential promoters for use in RGCs. We proceeded to evaluate *in vivo* one of these, *Nefh*, a putative promoter sequence that showed significant conservation between species, high retina expression and RGC enrichment and that was of a suitable size for use in AAV-mediated gene delivery vectors. We established that the *Nefh* upstream sequence efficiently drives expression in RGCs following intravitreal injection of AAV.*Nefh*-EGFP.

Following intravitreal delivery of either AAV*.Nefh-*EGFP or AAV.*CMV-*EGFP, EGFP expression patterns were compared by histology. Serotype AAV2/2 was chosen both for its efficient transduction of mouse RGCs, as well as its use and tolerance in the human eye, as has been observed in several clinical trials (Zhang et al., 2009; Busskamp et al., 2010; Koilkonda et al., 2014; MacLaren et al., 2014; Bennett et al., 2016; Ghazi et al., 2016; Sengupta et al., 2016; Yang et al., 2016). Both the *Nefh* and *CMV* promoters drove effective expression of EGFP in the GCL (Figure 3). Of note, the AAV.*CMV-*EGFP vector also resulted in expression in the INL, while AAV.*Nefh*-EGFP expression was predominantly confined to the GCL, with few EGFP positive cells observed in the INL (Figures 3, 5A). Furthermore, when an increased dose of the AAV.*Nefh*-EGFP vector was administered, the levels of EGFP expression in the INL did not increase, highlighting the relative specificity of the *Nefh* promoter compared to *CMV*.

Fifty percent of the GCL is composed of amacrine cells (Jeon et al., 1998; Akopian et al., 2016; webvision.med.utah.edu). Analysis of EGFP expression in Brn3a-negative cells, as well as in GABA-positive or ChAT-positive amacrine cells, two major types of amacrine cells in the mouse GCL, demonstrated that AAV*.Nefh-*EGFP resulted in transgene expression in significantly fewer amacrine cells compared to AAV*.CMV-*EGFP. While expression from the *Nefh* promoter was significantly restricted to ChAT-positive amacrine cells in the GCL compared to the *CMV* promoter, expression from both promoters were similar for GABA expressing amacrine cells in the GCL. This further highlights the relative specificity of the *Nefh* promoter sequence in targeting RGCs, and underlines its potential use for gene delivery to RGCs and its value for future gene therapies directed toward the retinal GCL. Of note, no significant difference was found between the numbers of transduced RGCs between the two doses of AAV.*Nefh*-EGFP. Previous studies have shown that only 40–60% of cells in the GCL are actually RGCs (Xiang et al., 1996; Schlamp et al., 2013); it may be that saturation of RGC transduction is being reached even at the lower AAV.*Nefh*-EGFP dose.

RGCs represent a heterogeneous population thought to comprise in the region of 30 discrete types (Baden et al., 2016), which together represent approximately 1% of cells in the retina. This has made isolation of pure populations of RGCs highly challenging within the field. Methods that have traditionally been used to enrich for RGCs, commonly using a Thy1 antibody, have included immunopanning (Barres et al., 1988), density gradient centrifugation (Kornguth et al., 1981), and magnetic cell separation (Shoge et al., 1999). More recently flow cytometry-based methods with the Thy1.2 antibody have been used for RGC enrichment (Chintalapudi et al., 2016). These studies have highlighted that while the Thy1 antibody does indeed enrich for RGCs it does not exclusively label these cells, indicating that RGC-isolation methodologies still require optimization. In the current study we used Thy1.2-based flow cytometry to support the data from immunohistochemistry. Similar to other studies, we found that the antibody did not exclusively isolate RGCs, based on the percentage of Thy1-positive cells. However, in addition we confirmed at the RNA level that *Thy1* was enriched in our flow-sorted population. Using flow cytometry we demonstrated enrichment of Thy1-positive cells within EGFP-positive cell populations to be greater in AAV*.Nefh-*EGFP vs. AAV*.CMV-*EGFP treated retinal cell samples. This confirms our histological data, and indicates preferential gene expression in RGCs with the *Nefh* promoter.

The purpose of this study was two-fold, involving identification of candidate RGC promoters for potential use in AAV-mediated gene therapies, and moreover the validation of the utilized methodology for characterisation of putative promoter sequences (Figures 1, 2). As sequencing costs continue to decrease, and techniques such as RNAseq become more widely adopted, access to transcriptomic datasets from a wide variety of cell types will become more readily available. The availability of such large datasets will be a powerful resource, which, in a similar fashion to the present work, could be exploited to identify, characterize and validate promoter sequences. The current study utilized an AAV2/2 vector to facilitate the transduction of mouse RGCs. However, it has been previously observed that, while AAV2/2 is well tolerated in the human eye when administered subretinally, its transduction efficiency in primate RGCs is inferior to that of mice (Ivanova et al., 2010; Yin et al., 2011; Tshilenge et al., 2016). The development of new capsid serotypes such as AAV8BP2 (Ramachandran et al., 2016), or new methods of administering AAV2/2 (as in the sub-ILM delivery of Boye et al., 2016) should aid in addressing this. It is important to note that the repertoire of AAV serotypes available for gene delivery is rapidly increasing. These improved viral capsids and delivery methods may increase the probability of detecting potential off-target transcriptional activity from tissue-specific promoters, including *Nefh*, beyond what was seen in the current study employing AAV2.

Intravitreal injection represents a route of vector administration that enables efficient transduction of RGCs. RGCs are the primary target cell population for gene therapies for many disorders including Leber Hereditary Optic Neuropathy (LHON), dominant optic atrophy (DOA), glaucoma and the retinal endophenotypes that are a feature of many neurodegenerative disorders, such as multiple sclerosis (Farrar et al., 2013). While intravitreal administration provides access to RGCs, it may more readily result in stimulating immune response(s) to vectors such as AAV compared to subretinal administration (Li et al., 2008). It would therefore be valuable to minimize the therapeutic vector dose, and to confine transgene expression to the target cells of interest, thereby limiting undesired side effects.

Furthermore, observations regarding patterns of cellular loss in end stage photoreceptor degenerations have highlighted the retention of certain retinal layers. While frequently the photoreceptor layer degenerates, many other retinal cells remain relatively intact, including bipolar, amacrine, horizontal and RGCs. These observations have been elegantly juxtaposed with the identification of light sensitive molecules from organisms such as algae and archaebacteria. Optogenetics is the expression of these molecules, provided as a gene therapy or protein, in non-light sensitive neurons thereby introducing a capacity for light detection. RGCs represent one key target cell population for optogenetics (Farrar et al., 2014; Gaub et al., 2014), and hence the *Nefh* promoter characterized in the current study, in principle, may also be of value in the design of future optogenetic-based gene therapies for IRDs. The above highlights the potential utility of the *Nefh* promoter sequence identified in the current study providing preferential transgene expression in RGCs in the design of future gene therapies for many disorders involving RGCs. The study identifies a unique sequence within the *Nefh* upstream region, which should be of immense value in future gene therapies where preferential transgene expression in RGCs is desired.

## Ethics statement

Ethical approval for this work was provided by the Irish Medicines Board (now Health Products Regulatory Authority) pursuant to the European Union (Protection of Animals Used for Scientific Purposes) Regulations 2012 (SI 543/2012). All work was submitted to and approved by the Animal Research Ethics Committee (AREC) of Trinity College Dublin.

## Author contributions

KH: experimental design, experimentation, bioinformatics analysis, flow cytometry analysis, figures, writing and reviewing manuscript. NC: flow cytometry analysis, experimental design, experimentation, vector production, writing and reviewing manuscript. AP: flow cytometry analysis, experimentation, experimental design, histological analysis, figures, writing and reviewing manuscript. AB: flow cytometry analysis and sorting, supplementary figures, reviewing manuscript. PH: writing and reviewing manuscript. PK: Intravitreal injections, reviewing manuscript. SM: flow cytometry analysis, project design, experimental design, experimentation, writing and reviewing manuscript. GF: PI, project design, experimental design, writing and reviewing manuscript.

### Conflict of interest statement

The authors have filed a patent describing aspects of retinal ganglion cell promoter(s).

## Supplementary material

Figure S1 Flow cytometry analysis of *CMV* and *Nefh* promoter mediated EGFP expression. Eyes were injected intravitreally with either AAV.*CMV*-EGFP (3 × 10^9^) or AAV.*Nefh*-EGFP (9 × 10^9^vg). Three weeks post-injection, retinas were dissociated and processed for flow cytometry analysis, using a Thy1 antibody conjugated to PE-Cy5. Nucleated cell populations were identified on the basis of DRAQ5 positive labeling (data not shown) and forward (FSC) and side (SSC) scatter **(a)**, and singlets identified **(b,c)**. Thy1 (x-axis) and EGFP (y-axis) gates were created based on wildtype retinas that had not been treated with Thy1 antibody and wildtype retinas that had been treated with Thy1 antibody, representing Thy1-negative **(d)** and Thy1-positive **(e)** control samples. Using these pre-defined gates transduced retinal samples (*n* = 6 per group) were sorted against EGFP and PE-Cy5 (Thy1; **f,g**). Percentage of cells in each quadrant are indicated. Enrichment values were generated by dividing the percentage of Thy1 and EGFP double positive cells by the percentage of EGFP-positive Thy1-negative cells. Thy1 positive cells (from *n* = 12 retinal samples) and non-labeled singlets with a similar FFC/SSC profile (from *n* = 9 retinal samples) were collected and pooled and *Thy1* mRNA levels were established by RT-QPCR **(h)**. *Thy1* mRNA enrichment in Thy1 antibody positive cells was calculated from the ΔCt value divided by the ratio of Thy1-positive cells to whole retinal cells.

Table S1 List of animal sequences used for conservation alignment. A placental mammal species alignment (phastConsElements60wayEuarchontoGlires) was used for the conservation alignment seen in Figure 2. Species are grouped as Glires, Primates, and other placental mammals, with species names, sequence assembly dates, and assembly details listed.

## References

[B1] Akopian A. Kumar S. Ramakrishnan H. Viswanathan S. Bloomfield S. A. (2016). Amacrine cells coupled to ganglion cells via gap junctions are highly vulnerable in glaucomatous mouse retinas. J. Comp. Neurol. [Epub ahead of print]. 10.1002/cne.24074 27411041 PMC7047713

[B2] Alić I. Kosi N. Kapuralin K. Gorup D. Gajović S. Pochet R.. (2016). Neural stem cells from mouse strain Thy1 YFP-16 are a valuable tool to monitor and evaluate neuronal differentiation and morphology. Neurosci. Lett. 634, 32–41. 10.1016/j.neulet.2016.10.001 27712955

[B3] Baden T. Berens P. Franke K. Román Rosón M. Bethge M. Euler T. (2016). The functional diversity of retinal ganglion cells in the mouse. Nature 529, 345–350. 10.1038/nature16468 26735013 PMC4724341

[B4] Bainbridge J. W. B. Mehat M. S. Sundaram V. Robbie S. J. Barker S. E. Ripamonti C.. (2015). Long-Term Effect of Gene Therapy on Leber's Congenital Amaurosis. N. Engl. J. Med. 372, 1887–1897. 10.1056/NEJMoa1414221 25938638 PMC4497809

[B5] Bainbridge J. W. B. Smith A. J. Barker S. S. Robbie S. Henderson R. Balaggan K.. (2008). Effect of gene therapy on visual function in Leber's congenital amaurosis. N. Engl. J. Med. 358, 2231–2239. 10.1056/NEJMoa0802268 18441371

[B6] Barres B. A. Silverstein B. E. Corey D. R. Chun L. L. Y. (1988). Immunological, morphological, and electrophysiological variation among retinal ganglion cells purified by panning. Neuron 1, 791–803. 10.1016/0896-6273(88)90127-4 2908449

[B7] Bennett J. Wellman J. Marshall K. A. McCague S. Ashtari M. DiStefano-Pappas J.. (2016). Safety and durability of effect of contralateral-eye administration of AAV2 gene therapy in patients with childhood-onset blindness caused by RPE65 mutations: a follow-on phase 1 trial. Lancet 388, 661–672. 10.1016/S0140-6736(16)30371-3 27375040 PMC5351775

[B8] Bennett J. Zeng Y. Bajwa R. Klatt L. Li Y. Maguire A. M. (1998). Adenovirus-mediated delivery of rhodopsin-promoted bcl-2 results in a delay in photoreceptor cell death in the rd/rd mouse. Gene Ther. 5, 1156–1164. 10.1038/sj.gt.3300733 9930315

[B9] Bessant D. A. R. Ali R. R. Bhattacharya S. S. (2001). Molecular genetics and prospects for therapy of the inherited retinal dystrophies. Curr. Opin. Genet. Dev. 11, 307–316. 10.1016/S0959-437X(00)00195-7 11377968

[B10] Boye S. E. Alexander J. J. Witherspoon C. D. Boye S. L. Peterson J. J. Clark M. E.. (2016). Highly efficient delivery of adeno-associated viral vectors to the primate retina. Hum. Gene Ther. 27, 580–597. 10.1089/hum.2016.085 27439313 PMC4991591

[B11] Boye S. E. Boye S. L. Pang J. Ryals R. Everhart D. Umino Y.. (2010). Functional and behavioral restoration of vision by gene therapy in the guanylate cyclase-1 (GC1) knockout mouse. PLoS ONE 5:11306. 10.1371/journal.pone.0011306 20593011 PMC2892468

[B12] Busskamp V. Duebel J. Balya D. Fradot M. Viney T. J. Siegert S.. (2010). Genetic reactivation of cone photoreceptors restores visual responses in retinitis pigmentosa. Science 329, 413–417. 10.1126/science.1190897 20576849

[B13] Callaway E. M. (2005). Structure and function of parallel pathways in the primate early visual system. J. Physiol. 566, 13–19. 10.1113/jphysiol.2005.088047 15905213 PMC1464718

[B14] Chadderton N. Palfi A. Millington-Ward S. Gobbo O. Overlack N. Carrigan M.. (2013). Intravitreal delivery of AAV-NDI1 provides functional benefit in a murine model of Leber hereditary optic neuropathy. Eur. J. Hum. Genet. 21, 62–68. 10.1038/ejhg.2012.112 22669418 PMC3522193

[B15] Chintalapudi S. R. Djenderedjian L. Stiemke A. B. Steinle J. J. Jablonski M. M. Morales-Tirado V. M. (2016). Isolation and molecular profiling of primary mouse retinal ganglion cells: Comparison of phenotypes from healthy and glaucomatous retinas. Front. Aging Neurosci. 8:93. 10.3389/fnagi.2016.00093 27242509 PMC4870266

[B16] Choi V. W. Bigelow C. E. McGee T. L. Gujar A. N. Li H. Hanks S. M.. (2015). AAV-mediated RLBP1 gene therapy improves the rate of dark adaptation in Rlbp1 knockout mice. Mol. Ther. Methods Clin. Dev. 2:15022. 10.1038/mtm.2015.22 26199951 PMC4495722

[B17] Choudhury S. Strang C. E. Alexander J. J. Scalabrino M. L. Lynch Hill J. Kasuga D. T.. (2016). Novel Methodology for Creating Macaque Retinas with Sortable Photoreceptors and Ganglion Cells. Front. Neurosci. 10:551. 10.3389/fnins.2016.00551 27990105 PMC5131003

[B18] Cronin T. Vandenberghe L. H. Hantz P. Juttner J. Reimann A. Kacsó A.-E.. (2014). Efficient transduction and optogenetic stimulation of retinal bipolar cells by a synthetic adeno-associated virus capsid and promoter. EMBO Mol. Med. 6, 1–16. 10.15252/emmm.201404077 25092770 PMC4197864

[B19] Crooks J. Kolb H. (1992). Localization of GABA, glycine, glutamate and tyrosine hydroxylase in the human retina. J. Comp. Neurol. 315, 287–302. 10.1002/cne.903150305 1346792

[B20] Dalkara D. Byrne L. C. Klimczak R. R. Visel M. Yin L. Merigan W. H.. (2013). *In vivo*-directed evolution of a new adeno-associated virus for therapeutic outer retinal gene delivery from the vitreous. Sci. Transl. Med. 5:189ra76. 10.1126/scitranslmed.3005708 23761039

[B21] Diester I. Kaufman M. T. Mogri M. Pashaie R. Goo W. Yizhar O.. (2011). An optogenetic toolbox designed for primates. Nat. Neurosci. 14, 387–397. 10.1038/nn.2749 21278729 PMC3150193

[B22] Dong J. Y. Fan P. D. Frizzell R. A. (1996). Quantitative analysis of the packaging capacity of recombinant adeno-associated virus. Hum. Gene Ther. 7, 2101–2112. 10.1089/hum.1996.7.17-2101 8934224

[B23] Farrar G. J. Carrigan M. Dockery A. Millington-Ward S. Palfi A. Chadderton N.. (2017). Toward an elucidation of the molecular genetics of inherited retinal degenerations. Hum. Mol. Genet. 26, R2–R11. 10.1093/hmg/ddx185 28510639 PMC5886474

[B24] Farrar G. J. Chadderton N. Kenna P. F. Millington-Ward S. (2013). Mitochondrial disorders: aetiologies, models systems, and candidate therapies. Trends Genet. 29, 488–497. 10.1016/j.tig.2013.05.005 23756086

[B25] Farrar G. J. Millington-Ward S. Chadderton N. Mansergh F. C. Palfi A. (2014). Gene therapies for inherited retinal disorders. Vis. Neurosci. 31, 289–307. 10.1017/S0952523814000133 24949856

[B26] Feuer W. J. Schiffman J. C. Davis J. L. Porciatti V. Gonzalez P. Koilkonda R. D.. (2016). Gene therapy for leber hereditary optic neuropathy: initial Results. Ophthalmology 123, 558–570. 10.1016/j.ophtha.2015.10.025 26606867 PMC5287094

[B27] Flannery J. G. Zolotukhin S. Vaquero M. I. LaVail M. M. Muzyczka N. Hauswirth W. W. (1997). Efficient photoreceptor-targeted gene expression *in vivo* by recombinant adeno-associated virus. Proc. Natl. Acad. Sci. U.S.A. 94, 6916–6921. 10.1073/pnas.94.13.6916 9192666 PMC21259

[B28] Gaub B. M. Berry M. H. Holt A. E. Reiner A. Kienzler M. A. Dolgova N.. (2014). Restoration of visual function by expression of a light-gated mammalian ion channel in retinal ganglion cells or ON-bipolar cells. Proc. Natl. Acad. Sci. U.S.A. 111, E5574–E5583. 10.1073/pnas.1414162111 25489083 PMC4280620

[B29] Ghazi N. G. Abboud E. B. Nowilaty S. R. Alkuraya H. Alhommadi A. Cai H.. (2016). Treatment of retinitis pigmentosa due to MERTK mutations by ocular subretinal injection of adeno-associated virus gene vector: results of a phase I trial. Hum. Genet. 135, 327–343. 10.1007/s00439-016-1637-y 26825853

[B30] Grieger J. C. Samulski R. J. (2005). Packaging capacity of adeno-associated virus serotypes: impact of larger genomes on infectivity and postentry steps. J. Virol. 79, 9933–9944. 10.1128/JVI.79.15.9933-9944.2005 16014954 PMC1181570

[B31] Hauswirth W. W. Aleman T. S. Kaushal S. Cideciyan A. V. Schwartz S. B. Wang L.. (2008). Treatment of leber congenital amaurosis due to RPE65 mutations by ocular subretinal injection of adeno-associated virus gene vector: short-term results of a phase I trial. Hum. Gene Ther. 19, 979–990. 10.1089/hum.2008.107 18774912 PMC2940541

[B32] Ivanova E. Hwang G. S. Pan Z. H. Troilo D. (2010). Evaluation of AAV-mediated expression of chop2-GFP in the marmoset retina. Investig. Ophthalmol. Vis. Sci. 51, 5288–5296. 10.1167/iovs.10-5389 20484599 PMC2939198

[B33] Jeon C. J. Strettoi E. Masland R. H. (1998). The major cell populations of the mouse retina. J. Neurosci. 18, 8936–8946. 9786999 10.1523/JNEUROSCI.18-21-08936.1998 PMC6793518

[B34] Kay C. N. Ryals R. C. Aslanidi G. V. Min S. H. Ruan Q. Sun J.. (2013). Targeting Photoreceptors via Intravitreal Delivery Using Novel, Capsid-Mutated AAV Vectors. PLoS ONE 8:62097. 10.1371/journal.pone.0062097 23637972 PMC3637363

[B35] Kent W. J. Sugnet C. W. Furey T. S. Roskin K. M. Pringle T. H. Zahler A. M.. (2002). The human genome browser at UCSC. Genome. Res. 12, 996–1006. 10.1101/gr.229102 12045153 PMC186604

[B36] Khani S. C. Pawlyk B. S. Bulgakov O. V. Kasperek E. Young J. E. Adamian M.. (2007). AAV-mediated expression targeting of rod and cone photoreceptors with a human rhodopsin kinase promoter. Investig. Ophthalmol. Vis. Sci. 48, 3954–3961. 10.1167/iovs.07-0257 17724172

[B37] Kim C. Y. Kuehn M. H. Clark A. F. Kwon Y. H. (2006). Gene expression profile of the adult human retinal ganglion cell layer. Mol. Vis. 12, 1640–1648. Available online at: www.molvis.org 17200664

[B38] Koilkonda R. D. Chou T.-H. Porciatti V. Hauswirth W. W. Guy J. (2010). Induction of rapid and highly efficient expression of the human ND4 complex I subunit in the mouse visual system by self-complementary adeno-associated virus. Arch. Ophthalmol. 128, 876–883. 10.1001/archophthalmol.2010.135 20625049 PMC3431796

[B39] Koilkonda R. D. Yu H. Chou T.-H. Feuer W. J. Ruggeri M. Porciatti V.. (2014). Safety and effects of the vector for the leber hereditary optic neuropathy gene therapy clinical trial. JAMA Ophthalmol. 33136, 409–420. 10.1001/jamaophthalmol.2013.7630 24457989 PMC4266107

[B40] Kornguth S. Auerbach R. Grieves J. Kahan L. (1981). Immunological reactivity of monoclonal antibodies prepared against large ganglion cells from bovine retina. Neurosci. Lett. 27, 151–157. 10.1016/0304-3940(81)90260-3 7322449

[B41] Kügler S. Kilic E. Bähr M. (2003a). Human synapsin 1 gene promoter confers highly neuron-specific long-term transgene expression from an adenoviral vector in the adult rat brain depending on the transduced area. Gene Ther. 10, 337–347. 10.1038/sj.gt.3301905 12595892

[B42] Kügler S. Lingor P. Schöll U. Zolotukhin S. Bähr M. (2003b). Differential transgene expression in brain cells *in vivo* and *in vitro* from AAV-2 vectors with small transcriptional control units. Virology 311, 89–95. 10.1016/S0042-6822(03)00162-4 12832206

[B43] Lebherz C. Maguire A. Tang W. Bennett J. Wilson J. M. (2008). Novel AAV serotypes for improved ocular gene transfer. J. Gene Med. 10, 375–382. 10.1002/jgm.1126 18278824 PMC2842078

[B44] Li Q. Miller R. Han P.-Y. Pang J. Dinculescu A. Chiodo V.. (2008). Intraocular route of AAV2 vector administration defines humoral immune response and therapeutic potential. Mol. Vis. 14, 1760–1769. Available online at: www.molvis.org 18836574 PMC2559816

[B45] Lopez A. J. Kramar E. Matheos D. P. White A. O. Kwapis J. Vogel-Ciernia A.. (2016). Promoter-specific effects of DREADD modulation on hippocampal synaptic plasticity and memory formation. J. Neurosci. 36, 3588–3599. 10.1523/JNEUROSCI.3682-15.2016 27013687 PMC4804014

[B46] MacLaren R. E. Groppe M. Barnard A. R. Cottriall C. L. Tolmachova T. Seymour L.. (2014). Retinal gene therapy in patients with choroideremia: initial fi ndings from a phase 1/2 clinical trial. Lancet 383, 1129–1137. 10.1016/S0140-6736(13)62117-0 24439297 PMC4171740

[B47] Mao Y. Wang X. Yan R. Hu W. Li A. Wang S.. (2016). Single point mutation in adeno-associated viral vectors -DJ capsid leads to improvement for gene delivery *in vivo*. BMC Biotechnol. 16:1. 10.1186/s12896-015-0230-0 26729248 PMC4700607

[B48] Millington-Ward S. Chadderton N. O'Reilly M. Palfi A. Goldmann T. Kilty C.. (2011). Suppression and replacement gene therapy for autosomal dominant disease in a murine model of dominant retinitis pigmentosa. Mol. Ther. 19, 642–649. 10.1038/mt.2010.293 21224835 PMC3070095

[B49] Molday L. L. Djajadi H. Yan P. Szczygiel L. Boye S. L. Chiodo V. A.. (2013). RD3 gene delivery restores guanylate cyclase localization and rescues photoreceptors in the Rd3 mouse model of leber congenital amaurosis 12. Hum. Mol. Genet. 22, 3894–3905. 10.1093/hmg/ddt244 23740938 PMC3766183

[B50] Mowat F. M. Gornik K. R. Dinculescu A. Boye S. L. Hauswirth W. W. Petersen-Jones S. M.. (2014). Tyrosine capsid-mutant AAV vectors for gene delivery to the canine retina from a subretinal or intravitreal approach. Gene Ther. 21, 96–105. 10.1038/gt.2013.64 24225638 PMC3880610

[B51] Mueller C. Flotte T. R. (2008). Clinical gene therapy using recombinant adeno-associated virus vectors. Gene Ther. 15, 858–863. 10.1038/gt.2008.68 18418415

[B52] Nadal-Nicolás F. M. Jiménez-López M. Sobrado-Calvo P. Nieto-López L. Cánovas-Martinez I. Salinas-Navarro M.. (2009). Brn3a as a marker of retinal ganglion cells: qualitative and quantitative time course studies in naïve and optic nerve-injured retinas. Investig. Ophthalmol. Vis. Sci. 50, 3860–3868. 10.1167/iovs.08-3267 19264888

[B53] Nadal-Nicolás F. M. Salinas-Navarro M. Jiménez-López M. Sobrado-Calvo P. Villegas-Pérez M. P. Vidal-Sanz M.. (2014). Displaced retinal ganglion cells in albino and pigmented rats. Front. Neuroanat. 8:99. 10.3389/fnana.2014.00099 25339868 PMC4186482

[B54] O'Reilly M. Palfi A. Chadderton N. Millington-Ward S. Ader M. Cronin T.. (2007). RNA interference–mediated suppression and replacement of human rhodopsin *in vivo*. Am. J. Hum. Genet. 81, 127–135. 10.1086/519025 17564969 PMC1950918

[B55] Palfi A. Chadderton N. McKee A. G. Blanco Fernandez A. Humphries P. Kenna P. F.. (2012). Efficacy of codelivery of dual AAV2/5 vectors in the murine Retina and Hippocampus. Hum. Gene Ther. 23, 847–858. 10.1089/hum.2011.142 22545762

[B56] Palfi A. Chadderton N. O'Reilly M. Nagel-Wolfrum K. Wolfrum U. Bennett J.. (2015). Efficient gene delivery to photoreceptors using AAV2/rh10 and rescue of the Rho(-/-) mouse. Mol. Ther. Methods Clin. Dev. 2:15016. 10.1038/mtm.2015.16 26029727 PMC4444994

[B57] Palfi A. Millington-Ward S. Chadderton N. O'Reilly M. Goldmann T. Humphries M. M.. (2010). Adeno-associated virus-mediated rhodopsin replacement provides therapeutic benefit in mice with a targeted disruption of the rhodopsin gene. Hum. Gene Ther. 21, 311–323. 10.1089/hum.2009.119 19824806

[B58] Petrs-Silva H. Dinculescu A. Li Q. Min S.-H. Chiodo V. Pang J.-J.. (2009). High-efficiency transduction of the mouse retina by tyrosine-mutant AAV serotype vectors. Mol. Ther. 17, 463–471. 10.1038/mt.2008.269 19066593 PMC2835095

[B59] Ramachandran P. Lee V. Wei Z. Song J. Y. Casal G. Cronin T.. (2016). Evaluation of dose and safety of AAV7m8 and AAV8BP2 in the non-human primate retina. Hum. Gene Ther. Hum. 2016:111. 10.1089/hum.2016.111 27750461 PMC5312498

[B60] Rousso D. L. Qiao M. Kagan R. D. Yamagata M. Palmiter R. D. Sanes J. R. (2016). Two Pairs of ON and OFF Retinal Ganglion Cells Are Defined by Intersectional Patterns of Transcription Factor Expression. Cell Rep. 15, 1930–1944. 10.1016/j.celrep.2016.04.069 27210758 PMC4889540

[B61] Sanes J. R. Masland R. H. (2014). The types of retinal ganglion cells: current status and implications for neuronal classification. Annu. Rev. Neurosci. 38:150421150146009. 10.1146/annurev-neuro-071714-034120 25897874

[B62] Schlamp C. L. Montgomery A. D. Mac Nair C. E. Schuart C. Willmer D. J. Nickells R. W. (2013). Evaluation of the percentage of ganglion cells in the ganglion cell layer of the rodent retina. Mol. Vis. 19, 1387–1396. Available online at: www.molvis.org 23825918 PMC3695759

[B63] Sengupta A. Chaffiol A. Macé E. Caplette R. Desrosiers M. Lampič M.. (2016). Red-shifted channelrhodopsin stimulation restores light responses in blind mice, macaque retina, and human retina. EMBO Mol. Med. 8, 1248–1264. 10.15252/emmm.201505699 27679671 PMC5090658

[B64] Shoge K. Mishima H. K. Mukai S. Shinya M. Ishihara K. Kanno M.. (1999). Rat retinal ganglion cells culture enriched with the magnetic cell sorter. Neurosci. Lett. 259, 111–114. 10025570 10.1016/s0304-3940(98)00918-5

[B65] Spanopoulou E. Giguere V. Grosveld F. (1991). The functional domains of the murine Thy-1 gene promoter. Mol. Cell. Biol. 11, 2216–2228. 10.1128/MCB.11.4.2216.Updated 1672442 PMC359917

[B66] Struebing F. L. Lee R. K. Williams R. W. Geisert E. E. (2016). Genetic networks in mouse retinal ganglion cells. Front. Genet. 7, 1–14. 10.3389/fgene.2016.00169 27733864 PMC5039302

[B67] Sun L. O. Brady C. M. Cahill H. Al-Khindi T. Sakuta H. Dhande O. S.. (2015). Functional assembly of accessory optic system circuitry critical for compensatory eye movements. Neuron 86, 971–984. 10.1016/j.neuron.2015.03.064 25959730 PMC4441577

[B68] Sun X. Pawlyk B. Xu X. Liu X. Bulgakov O. V. Adamian M.. (2010). Gene therapy with a promoter targeting both rods and cones rescues retinal degeneration caused by AIPL1 mutations. Gene Ther. 17, 117–131. 10.1038/gt.2009.104 19710705 PMC2804971

[B69] Sweeney N. T. James K. N. Nistorica A. Lorig-Roach R. M. Feldheim D. A. (2017). Expression of transcription factors divide retinal ganglion cells into distinct classes. J. Comp. Neurol. 520, 633–655. 10.1002/cne.24172 28078709 PMC9444162

[B70] Trost A. Motloch K. Bruckner D. Schroedl F. Bogner B. Kaser-Eichberger A.. (2015). Time-dependent retinal ganglion cell loss, microglial activation and blood-retina-barrier tightness in an acute model of ocular hypertension. Exp. Eye Res. 136, 59–71. 10.1016/j.exer.2015.05.010 26001526

[B71] Tshilenge K.-T. Ameline B. Weber M. Mendes-Madeira A. Nedellec S. Biget M.. (2016). Vitrectomy before intravitreal injection of AAV2/2 vector promotes efficient transduction of retinal ganglion cells in dogs and nonhuman primates. Hum. Gene Ther. Methods 27, 122–134. 10.1089/hgtb.2016.034 27229628

[B72] Wässle H. Chun M. H. Müller F. (1987). Amacrine cells in the ganglion cell layer of the cat retina. J. Comp. Neurol. 265, 391–408. 10.1002/cne.902650308 3693612

[B73] Wert K. J. Davis R. J. Sancho-pelluz J. Nishina P. M. Tsang S. H. (2013). Gene therapy provides long-term visual function in a pre-clinical model of retinitis pigmentosa. Hum. Mol. Genet. 22, 558–567. 10.1093/hmg/dds466 23108158 PMC3542865

[B74] Xiang M. Zhou H. Nathans J. (1996). Molecular biology of retinal ganglion cells. Proc. Natl. Acad. Sci. U.S.A. 93, 596–601. 8570601 10.1073/pnas.93.2.596 PMC40097

[B75] Yang S. Ma S.-Q. Wan X. He H. Pei H. Zhao M.-J.. (2016). Long-term outcomes of gene therapy for the treatment of Leber's hereditary optic neuropathy. EBioMedicine 10, 258–268. 10.1016/j.ebiom.2016.07.002 27426279 PMC5006665

[B76] Yin L. Greenberg K. Hunter J. J. Dalkara D. Kolstad K. D. Masella B. D.. (2011). Intravitreal injection of AAV2 transduces macaque inner retina. Investig. Ophthalmol. Vis. Sci. 52, 2775–2783. 10.1167/iovs.10-6250 21310920 PMC3088562

[B77] Zhang Y. Ivanova E. Bi A. Pan Z.-H. (2009). Ectopic expression of multiple microbial rhodopsins restores ON and OFF light responses in retinas with photoreceptor degeneration. J. Neurosci. 29, 9186–9196. 10.1523/JNEUROSCI.0184-09.2009 19625509 PMC2774241

[B78] Zhu Y. Xu J. Hauswirth W. W. DeVries S. H. (2014). Genetically targeted binary labeling of retinal neurons. J. Neurosci. 34, 7845–7861. 10.1523/JNEUROSCI.2960-13.2014 24899708 PMC4044247

