# Supplementary figures and images for: A Novel Retinal Ganglion Cell Promoter for Utility in AAV Vectors
Source: Front Neurosci. 2017 Sep 21;11:521. doi: 10.3389/fnins.2017.00521 (PMC5613148; doi:10.3389/fnins.2017.00521)

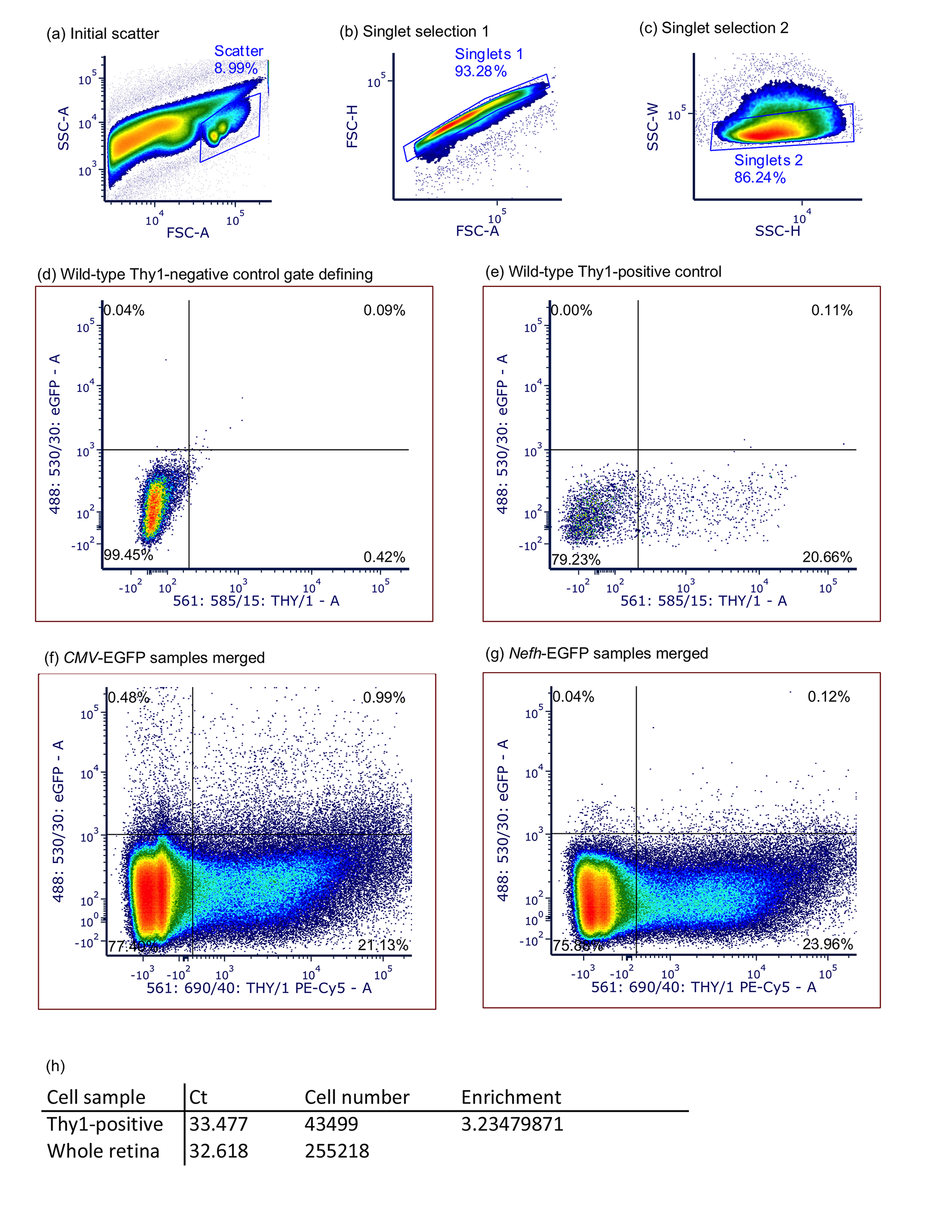

Supplement: Figure S1 — Flow cytometry analysis of CMV and Nefh promoter mediated EGFP expression. Eyes were injected intravitreally with either AAV.CMV-EGFP (3 × 10^9^) or AAV.Nefh-EGFP (9 × 10^9^vg). Three weeks post-injection, retinas were dissociated and processed for flow cytometry analysis, using a Thy1 antibody conjugated to PE-Cy5. Nucleated cell populations were identified on the basis of DRAQ5 positive labeling (data not shown) and forward (FSC) and side (SSC) scatter (a), and singlets identified (b,c). Thy1 (x-axis) and EGFP (y-axis) gates were created based on wildtype retinas that had not been treated with Thy1 antibody and wildtype retinas that had been treated with Thy1 antibody, representing Thy1-negative (d) and Thy1-positive (e) control samples. Using these pre-defined gates transduced retinal samples (n = 6 per group) were sorted against EGFP and PE-Cy5 (Thy1; f,g). Percentage of cells in each quadrant are indicated. Enrichment values were generated by dividing the percentage of Thy1 and EGFP double positive cells by the percentage of EGFP-positive Thy1-negative cells. Thy1 positive cells (from n = 12 retinal samples) and non-labeled singlets with a similar FFC/SSC profile (from n = 9 retinal samples) were collected and pooled and Thy1 mRNA levels were established by RT-QPCR (h). Thy1 mRNA enrichment in Thy1 antibody positive cells was calculated from the ΔCt value divided by the ratio of Thy1-positive cells to whole retinal cells. [file Image1.TIF]
