# Supplementary material for: A Novel Retinal Ganglion Cell Promoter for Utility in AAV Vectors
Source: Front Neurosci. 2017 Sep 21;11:521. doi: 10.3389/fnins.2017.00521 (PMC5613148; doi:10.3389/fnins.2017.00521)
Supplement: Table S1 — List of animal sequences used for conservation alignment. A placental mammal species alignment (phastConsElements60wayEuarchontoGlires) was used for the conservation alignment seen in Figure 2. Species are grouped as Glires, Primates, and other placental mammals, with species names, sequence assembly dates, and assembly details listed. [file Table1.DOCX]

Supplementary Material

A novel retinal ganglion cell promoter for utility in AAV vectors

Killian S. Hanlon^*1^, Naomi Chadderton^*1^, Arpad Palfi^1^, Peter Humphries^1^, Paul F. Kenna^1,2^, Sophia Millington-Ward^*1^, G. Jane Farrar^*1^

*** Correspondence:** Killian Hanlon: hanlonki@tcd.ie; G. Jane Farrar: jane.farrar@tcd.ie

# Supplementary Tables

| Animal | Species | Assembly Date | Assembly Name/details |
| --- | --- | --- | --- |
| Mouse | *Mus musculus* | Dec. 2011 | GRCm38/mm10 reference |
| Guinea pig | *Cavia porcellus* | Feb. 2008 | Broad/cavPor3 Syntenic net |
| Kangaroo rat | *Dipodomys ordii* | Jul. 2008 | Broad/dipOrd1 Reciprocal best |
| Naked mole-rat | *Heterocephalus glaber* | Jan. 2012 | Broad HetGla_female_1.0/hetGla2 Syntenic net |
| Pika | *Ochotona princeps* | Jul. 2008 | Broad/ochPri2 Reciprocal best |
| Rabbit | *Oryctolagus cuniculus* | Apr. 2009 | Broad/oryCun2 Syntenic net |
| Rat | *Rattus norvegicus* | Mar. 2012 | RGSC 5.0/rn5 Syntenic net |
| Squirrel | *Spermophilus tridecemlineatus* | Nov. 2011 | Broad/speTri2 Syntenic net |
|  |  |  |  |
| Tree shrew | *Tupaia belangeri* | Dec. 2006 | Broad/tupBel1 Reciprocal best |
| Marmoset | *Callithrix jacchus* | Mar. 2009 | WUGSC 3.2/calJac3 Syntenic net |
| Gorilla | *Gorilla gorilla* | May. 2011 | gorGor3 Syntenic net |
| Human | *Homo sapiens* | Feb. 2009 | GRCh37/hg19 Syntenic net |
| Mouse lemur | *Microcebus murinus* | Jun. 2003 | Broad/micMur1 Reciprocal best |
| Gibbon | *Nomascus leucogenys* | Jun. 2011 | GGSC Nleu1.1/nomLeu2 Syntenic net |
| Bushbaby | *Otolemur garnettii* | Mar. 2011 | Broad/otoGar3 Syntenic net |
| Chimp | *Pan troglodytes* | Feb. 2011 | Pan_troglodytes-2.1.4/panTro4 Syntenic net |
| Baboon | *Papio hamadryas* | Nov. 2008 | Baylor 1.0/papHam1 Reciprocal best |
| Orangutan | *Pongo pygmaeus abelii* | Jul. 2007 | WUGSC 2.0.2/ponAbe2 Syntenic net |
| Chinese rhesus | *Macaca mulatta* | Oct. 2010 | BGI CR_1.0/rheMac3 Syntenic net |
| Squirrel monkey | *Saimiri boliviensis* | Oct. 2011 | saiBol1 Syntenic net |
| Tarsier | *Tarsius syrichta* | Aug. 2008 | Broad/tarSyr1 Reciprocal best |
|  |  |  |  |
| Panda | *Ailuropoda melanoleuca* | Dec. 2009 | BGI-Shenzhen 1.0/ailMel1 Syntenic Net |
| Cow | *Bos taurus* | Oct. 2011 | Baylor Btau_4.6.1/bosTau7 Syntenic Net |
| Dog | *Canis lupus familiaris* | Sep. 2011 | Broad/canFam3 Syntenic net |
| Sloth | *Choloepus hoffmanni* | Jul. 2008 | Broad//choHof1 Reciprocal best |
| Armadillo | *Dasypus novemcinctus* | Dec. 2011 | Armadillo/dasNov3 Reciprocal best |
| Tenrec | *Echinops telfairi* | Jul. 2005 | Broad/echTel1 Reciprocal best |
| Horse | *Equus caballus* | Sep. 2007 | Broad/equCab2 Syntenic net |
| Hedgehog | *Erinaceus europaeus* | Jun. 2006 | Broad/eriEur1 Reciprocal best |
| Cat | *Felis catus* | Sep. 2011 | ISGSC Felis_catus 6.2/felCat5 Reciprocal best |
| Elephant | *Loxodonta africana* | Jul. 2009 | Broad/loxAfr3 Syntenic net |
| Microbat | *Myotis lucifugus* | Jul. 2010 | Broad/myoLuc2 Reciprocal best |
| Sheep | *Ovis aries* | Feb. 2010 | ISGC/oviAri1 Reciprocal best |
| Rock hyrax | *Procavia capensis* | Jul. 2008 | Broad/proCap1 Reciprocal best |
| Megabat | *Pteropus vampyrus* | Jul. 2008 | Broad/pteVam1 Reciprocal best |
| Shrew | *Sorex araneus* | Jun. 2006 | Broad/sorAra1 Reciprocal best |
| Pig | *Sus scrofa* | Aug. 2011 | SGSC Sscrofa10.2/susScr3 Syntenic net |
| Manatee | *Trichechus manatus latirostris* | Oct. 2011 | Broad v1.0/triMan1 Syntenic net |
| Dolphin | *Tursiops truncatus* | Oct. 2011 | Baylor Ttru_1.4/turTru2 Reciprocal best |
| Alpaca | *Vicugna pacos* | Jul. 2008 | Broad/vicPac1 Reciprocal best |

Table S1. List of animal sequences used for conservation alignment. A placental mammal species alignment (phastConsElements60wayEuarchontoGlires) was used for the conservation alignment seen in Figure 2. Species are grouped as Glires, Primates, and other placental mammals, with species names, sequence assembly dates, and assembly details listed.
